# Genetic determinants of SARS‐CoV‐2 and the clinical outcome of COVID‐19 in Southern Bangladesh

**DOI:** 10.1002/iid3.1171

**Published:** 2024-02-06

**Authors:** Md. Mahbub Hasan, Chayan Kumar Saha, H. M. Hamidullah Mehedi, Kallyan Chakma, Asma Salauddin, Md. Shakhawat Hossain, Farjana Sharmen, S. M. Rafiqul Islam, Afroza Akter Tanni, Farhana Yasmin, Al‐Shahriar Akash, Mohammad Enayet Hossain, Mojnu Miah, Sanjoy Kanti Biswas, Nahid Sultana, Adnan Mannan

**Affiliations:** ^1^ Department of Genetic Engineering and Biotechnology, Faculty of Biological Sciences University of Chittagong Chattogram Bangladesh; ^2^ Next Generation Sequencing, Research and Innovation Laboratory Chittagong (NRICh), Biotechnology Research and Innovation Center (BRIC) University of Chittagong Chattogram Bangladesh; ^3^ Bionamic AB Lund Sweden; ^4^ Department of Medicine 250 Bedded General Hospital Chattogram Bangladesh; ^5^ International Centre for Diarrhoeal Disease Research Bangladesh (icddr,b) Dhaka Bangladesh; ^6^ Department of Microbiology Chattogram Maa‐O‐Shishu Hospital Chattogram Bangladesh

**Keywords:** clade, clinical outcome, COVID‐19, genetic determinants, mutations, ORF1ab, S protein structure, SARS‐CoV‐2

## Abstract

**Background:**

The coronavirus disease 2019 (COVID‐19) pandemic has had a severe impact on population health. The genetic determinants of severe acute respiratory syndrome coronavirus 2 (SARS‐CoV‐2) in southern Bangladesh are not well understood.

**Methods:**

This study aimed to determine the genomic variation in SARS‐CoV‐2 genomes that have evolved over 2 years of the pandemic in southern Bangladesh and their association with disease outcomes and virulence of this virus. We investigated demographic variables, disease outcomes of COVID‐19 patients and genomic features of SARS‐CoV‐2.

**Results:**

We observed that the disease severity was significantly higher in adults (85.3%) than in children (14.7%), because the expression of angiotensin‐converting enzyme‐2 (ACE‐2) diminishes with ageing that causes differences in innate and adaptive immunity. The clade GK (*n* = 66) was remarkable between June 2021 and January 2022. Because of the mutation burden, another clade, GRA started a newly separated clustering in December 2021. The burden was significantly higher in GRA (1.5‐fold) highlighted in mild symptoms of COVID‐19 patients than in other clades (GH, GK, and GR). Mutations were accumulated mainly in S (22.15 mutations per segment) and ORF1ab segments. Missense (67.5%) and synonymous (18.31%) mutations were highly noticed in adult patients with mild cases rather than severe cases, especially in ORF1ab segments. Moreover, we observed many unique mutations in S protein in mild cases compared to severe, and homology modeling revealed that those might cause more folding in the protein's alpha helix and beta sheets.

**Conclusion:**

Our study identifies some risk factors such as age comorbidities (diabetes, hypertension, and renal disease) that are associated with severe COVID‐19, providing valuable insight regarding prioritizing vaccination for high‐risk individuals and allocating health care and resources. The findings of this work outlined the knowledge and mutational basis of SARS‐CoV‐2 for the next treatment steps. Further studies are needed to confirm the effects of structural and functional proteins of SARS‐CoV‐2 in detail for monitoring the emergence of new variants in future.

## Graphical abstract

The genetic determinants of severe acute respiratory syndrome coronavirus 2 in southern Bangladesh are not well understood. We investigated demographic variables, disease outcomes and genomic features of coronavirus disease 2019 patients. Missense (67.5%) and synonymous (18.31%) mutations and many unique mutations in S protein were highly noticed in adult patients with mild cases rather than severe cases.

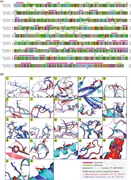

## 1 INTRODUCTION

The severe acute respiratory syndrome coronavirus 2 (SARS‐CoV‐2) is a highly pathogenic virus of the Coronaviridae family and has been identified as the etiological agent of the coronavirus disease 2019 (COVID‐19). Since its inception, the disease has caused mass mortality and active infections (https://covid19.who.int/). Currently, COVID‐19 is not being treated as a public health emergency, the formal declaration of which was made by the WHO in the first week of May 2023.^1^

SARS‐CoV‐2 is an enveloped, single‐stranded positive‐sense RNA virus with a genomic size of about 29.9 kb and 14 ORFs that encode 27 proteins, comprising 15 nonstructural, eight accessory, and four vital structural proteins. The first ORF (ORF1ab), responsible for translating polyproteins 1a and 1b, is found in two‐thirds of the viral RNA. The proteolytic cleavage of these proteins results in the formation of 15 nonstructural proteins. Four main structural proteins in SARS‐CoV‐2 are the spike glycoprotein (S), the membrane (M), the envelope (E), and the nucleocapsid (N).^2^, ^3^ Two functional subunits of S: S1and S2 play crucial roles in viral entrance into the host cell. The S1 subunit binds to the host cell receptor, while the S2 subunit mediates the fusion of the viral and host cell membranes.^4^

The single‐stranded RNA genome of SARS‐CoV‐2 mutates over time in community circulation, resulting in a different virus with distinct characteristics. Some of these mutations have altered the phase of disease severity, spread quickly to the community, and become public health concerns around the globe. Among most common mutations reported, the variants of concern (VOCs) include Alpha (B.1.1.7; in UK in December 2020),^5^ Delta (B.1.617.2; in India in October 2020),^6^ and Omicron (BA.1; in Botswana in November 2021).^7^

Mutations do not only have one‐sided outcomes for the virus but also put the strain under pressure sometimes. The mutation D614G found in VOCs Alpha, Beta, Gamma, and Delta has been shown to decrease the affinity of binding of spike protein with angiotensin‐converting enzyme‐2 (ACE2) receptor of the host by 1.5‐fold, while the N439K reduces the affinity by 3‐folds. In contrast, the N501Y mutation of the same protein makes it more susceptible to being attacked by antibodies developed by vaccinated people. It helps to avoid the severe manifestation of the disease.^8^, ^9^, ^10^, ^11^, ^12^ Modern genomic techniques used by researchers worldwide to understand viral pathogenesis are of utmost importance. Every day, more SARS‐CoV‐2 whole‐genome data from different parts of the world are submitted to publicly accessible databases like GISAID.^10^ During the COVID‐19 pandemic, genomic surveillance has been mandatory to understand the evolution of the disease pattern and necessary public health measures like geographical travel restrictions, vaccine development, and monitoring the effect of vaccines at the population level in real‐time.^13^

Understanding a larger picture of the association of COVID‐19 symptoms with comorbidities and biochemical markers from earlier research was impossible since some studies were not conducted for a large sample size.^14^ More research needs to be conducted on the long‐term problems among COVID‐19‐recovered patients. A good understanding of the clinical characterization and trends in the course of the disease may be gained by looking into the epidemiological traits of people in Bangladesh who have been diagnosed with COVID‐19.^15^ It should scrutinize these COVID‐19‐related factors and components to put effective prevention and treatment methods in place.

In this study, we investigated the genomic evolution of SARS‐CoV‐2 in the southern area of Bangladesh for about a 2‐year temporal extent as a cohort study. The aim was to decipher the role of the viral genome in its disease outcomes at a molecular level, which could help us understand how a virus‐causing pandemic evolves to a less virulent version. We used various techniques of molecular epidemiology, from clinical surveys to genomics, to examine the relationship between mutation frequency and three important patient characteristics—disease symptoms, gender, and age—to identify the genetic factors that influence the course of COVID‐19 outcomes. We emphasized the prevalent genetic determinants of SARS‐CoV‐2 that are likely to impact clinical outcomes in southern Bangladesh.

## 2 METHODS

### 2.1 Sample collection

A cross‐sectional study was conducted among COVID‐19‐positive patients confirmed by real‐time polymerase chain reaction (RT‐PCR) using nasopharyngeal and oropharyngeal swabs as clinical specimens. These COVID‐19‐diagnosed patients were admitted in two different hospitals in Bangladesh‐250 Bed General Hospital, Chattogram, and Chattogram Maa‐O‐Shishu Hospital. The patients were diagnosed positive for COVID‐19 on an average of 4.7 days (range 2–9 days) after the onset of clinical signs of SARS‐CoV‐2 positive through RT‐qPCR.

### 2.2 Case definition

The study population was categorized into two groups: mild and severe. Individuals who tested positive for SARS‐CoV‐2 but had no symptoms consistent with COVID‐19 or any of the various signs and symptoms of COVID‐19 (e.g., fever, cough, sore throat, malaise, headache, muscle pain, nausea, vomiting, diarrhea, loss of taste, and smell) but do not have shortness of breath, dyspnea, or abnormal chest imaging were considered as mild COVID‐19 patients. On the other side, individuals who had SpO_2_ < 94% on room air at sea level, a ratio of arterial partial pressure of oxygen to fraction of inspired oxygen (PaO_2_/FiO_2_) < 300 mmHg, a respiratory rate >30 breaths/min, or lung infiltrates >50% or had respiratory failure, septic shock, and/or multiple organ dysfunction were considered as severe COVID‐19 patients.

### 2.3 Sequencing

Samples collected from COVD‐19‐positive patients considering the clinical symptoms were selected for whole genome sequencing. Viral RNA extraction was performed from COVID‐19‐positive specimens using QIAamp viral RNA Mini Kit (Qiagen). First‐strand cDNA synthesis was performed using the LunaScript® RT SuperMix Kit (New England Biolabs (UK) Ltd), and second strand was synthesized using ARTIC nCoV‐2019 V3 primer panel and Q5® Hot Start High‐Fidelity 2× Master Mix (New England Biolabs (UK) Ltd) in two separate pools. In this step, non‐amplified single pool or both pools were excluded, and amplified DNA with the pool libraries were selected from library preparation where DNA amplification was confirmed through gel electrophoresis. After the dilution and merging of two pools, the nanopore sequencing libraries were prepared following the nCoV‐2019 sequencing protocol v3 (LoCost). Briefly, diluted amplicons were subjected to end‐prep reaction and multiplexed by native barcode (EXP‐NBD196). Barcoded amplicons were pooled based on the quantification report, and a 0.4x AMPure XP bead (Beckman Coulter) purification was carried out following the protocol. The libraries were quantified by the Qubit dsDNA High Sensitivity Assay Kit with a Qubit fluorometer (Invitrogen). The purified barcoded amplicon pools  taken forward to the sequencing adapter ligation step with ONT *Adapter Mix* II (*AMII*). The final library was sequenced on the FLO‐MIN106D flow cell on an ONT MinION MK 1C device. Real‐time base calling was performed using Guppy basecaller v.4.3.4 with the fast base calling mode. Generated FASTQ reads were quality‐checked, trimmed, and a consensus genome sequence was generated using the COVID‐19 assembly pipeline in EPI2ME platform, based on ARTIC Field Bioinformatics software (https://github.com/artic-network/fieldbioinformatics). The genomic sequences of surveyed COVID‐19 patients were submitted to the GISAID database (https://gisaid.org), which can be downloaded using the EPI_SET ID: EPI_SET_230225bd or doi: 10.55876/gis8.230225bd.

### 2.4 Mutation analysis

For mutation analysis, we employed an in‐built workflow in Python 3 and BASH programming languages (https://github.com/chayan7/variantFinder). The script uses the Minimap tool version 2.24‐r1122^16^ to align all the nucleotide sequences to the Wuhan SARS‐CoV‐2 virus reference genome (NC_045512.2). Then, SAMtools (v1.9)^17^ was used to sort and index the generated alignments. The bioinformatics program FreeBayes (v1.3.2) was later utilized for the variant calling process with this sorted alignment as the input.^18^ Finally, we used SnpEff v5.1d^19^ to annotate the variations and forecast their impact. We manually generated SnpEff databases for the annotation step using the reference genome annotation downloaded from the NCBI database.

### 2.5 Phylogenetic analysis

We uploaded metadata and sequencing data of all samples (*n* = 102) to GISAID. Subsequently, we aligned them using MAFFT v7.505^20^ through the Cipres Science Gateway v 3.3 portal. We then utilized RAxML^21^ with 100 bootstrap replicates to obtain a value (expressed as maximum likelihood bootstrap, MLB percentage) indicating the level of support for a particular branch in the tree topology based on the input alignment. For our Maximum Likelihood phylogenetic analysis, we selected the GTRCAT model of nucleotide substitution as it was determined to be the most suitable model for our data set.

### 2.6 Protein three‐dimensional structure prediction

We used the highly accurate deep learning algorithm AlphaFold2 to predict protein structure to understand how the mutation affects the protein structure and conformation.^22^ We chose one S protein representative from the severe and mild disease manifestation categories with the highest mutation burden for this analysis. Jalview was used to compare the predicted structures from the mild and severe categories with the reference S protein (YP_009724390.1).^23^

### 2.7 Statistical analysis

To visualize categorical data of different variables to see the trend of mutation and disease outcomes over time, we created an alluvial diagram using the open‐access web app RAWGraphs 2.0.^24^ Descriptive and inferential statistics were used to determine the correlation between COVID‐19 disease manifestation and demographic variables using IBM SPSS Statistics software (version 28; IBM UK Ltd). For this purpose, we mainly used the Pearson Chi‐Square tests described earlier.^25^ The relationship between demographic variables and mutation frequency per genome was tested using unpaired, nonparametric Mann–Whitney tests. The *p* = < .05 was considered significant and followed standard *p*‐value classification to assign asterisks. We used GraphPad Prism version 9.5.1 for visualizing the graphs (GraphPad Software). Shared symptoms among patients and comorbidities were visualized using Venn diagrams constructed using InteractiVenn.^26^

### 2.8 Ethical statement

The Institutional Review Board of the 250‐bed General Hospital, Chattogram, Bangladesh (IRB#00636) approved the protocol.

## 3 RESULTS

### 3.1 Demographic characteristics and disease outcomes

We enrolled a total of 102 COVID‐19 patients in this study. Based on the classification of the disease manifestation (either severe or mild), we scrutinized the relationship between demographic characteristics and the disease outcome, shading the variable of gender, age, case history of COVID‐19, vaccination history, patients' comorbidities, and hospitalization stage (Table 1). We found that gender and previous history of COVID‐19 did not directly contribute to the severity of the disease. However, we found that adults were more severely affected by the disease than children (85.3% vs. 14.7%; *p* = .021). Vaccination also helped to mitigate the manifestation of the disease significantly. Only one‐fourth of the participants suffered more severe outcomes than their nonvaccinated counterparts (17.6% vs. 82.4%; *p* = .008). Additionally, people with comorbidities had more severe and significant clinical outcomes from the disease (64.7% vs. 35.3%; *p* < .001).

**Table 1:** **Correlation between demographic characteristics of patients and disease outcomes of COVID‐19 in Chattogram (*n*** = **102)**.

Variable	Mild (*n* = 68)	Severe (*n* = 34)	Overall (*n* = 102)	*p*‐Value
Gender				
Female	30 (44.1%)	21 (61.82%)	51 (50%)	0.093
Male	38 (55.9%)	13 (38.25%)	51 (50%)	
Age group				
Adult	*43* (*63.2%)*	*29* (*85.3%)*	*72* (*70.6%)*	*0.021*
Children	*25* (*36.8%)*	*5* (*14.7%)*	*30* (*29.4%)*	
Previous history of COVID‐19				
No	62 (91.2%)	30 (88.2%)	92 (90.2%)	0.638
Yes	6 (8.8%))	4 (11.8%)	10 (9.8%)	
Previous history of vaccination				
No	38 (55.9%)	28 (82.4%)	66 (64.7%)	0.008
Yes	30 (44.1%)	6 (17.6%)	36 (35.3%)	
Comorbidities				
No	*50* (*73.5%)*	*12* (*35.3%)*	*62* (*60.8%)*	*<0.001*
Yes	*18* (*26.5%)*	*22* (*64.7%)*	*40* (*39.2%)*	
Hospitalization				
No	*55* (*80.9%)*	*3* (*8.8%)*	*58* (*55.9%)*	*<0.001*
Yes	*13* (*19.1%)*	*31* (*91.2%)*	*44* (*43.1%)*	

Abbreviation: COVID‐19, coronavirus disease 2019.

**Figure 1:**
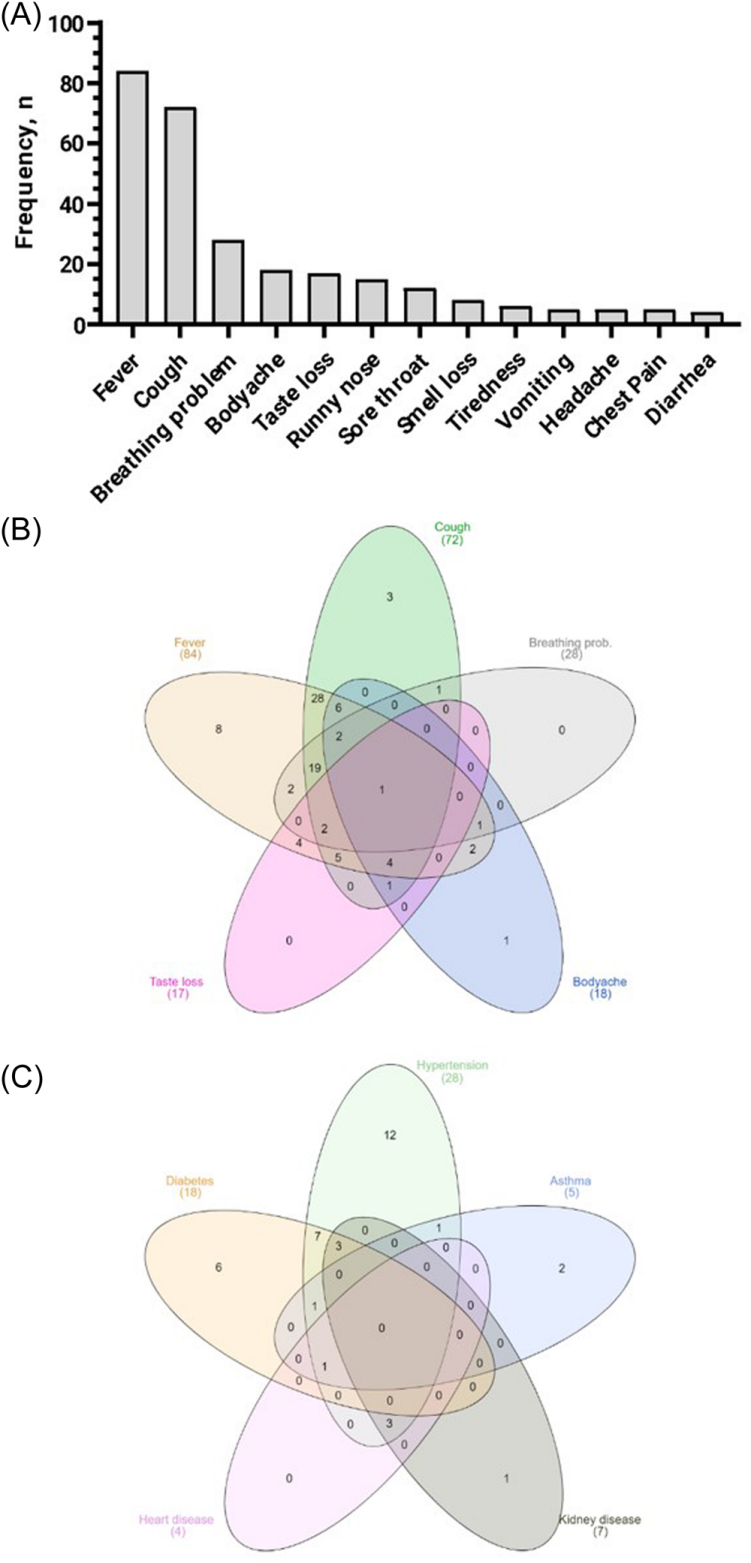
The manifestation of coronavirus disease 2019 (COVID‐19) among the participants of this study (*n* = 102) from Chittagong. Those include different symptoms reported (A), combinations of mostly occurring symptoms (B) and the distribution of frequently reported comorbidities among the participants in this study (C).

In our study, we recorded 13 symptoms among the patients with COVID‐19 (Figure 1A). Fever and cough were the most frequent symptoms. Other symptoms were breathing difficulty, sore throat, runny nose, loss of smell and taste, pains (from the body, chest, and headache), and even diarrhea. The most simultaneously reported symptoms were fever, cough, breathing problems and body aches (Figure 1B). Hypertension (*n* = 28) and diabetes (*n* = 18) were the most common comorbidities reported (Figure 1C). Only 12 patients experienced both comorbidities. We also recorded some other comorbidities like cardiovascular disease (CVD) (*n* = 2), cancer (*n* = 1), and Alzheimer's disease (*n* = 1).

### 3.2 Genomic diversity

**Figure 2:**
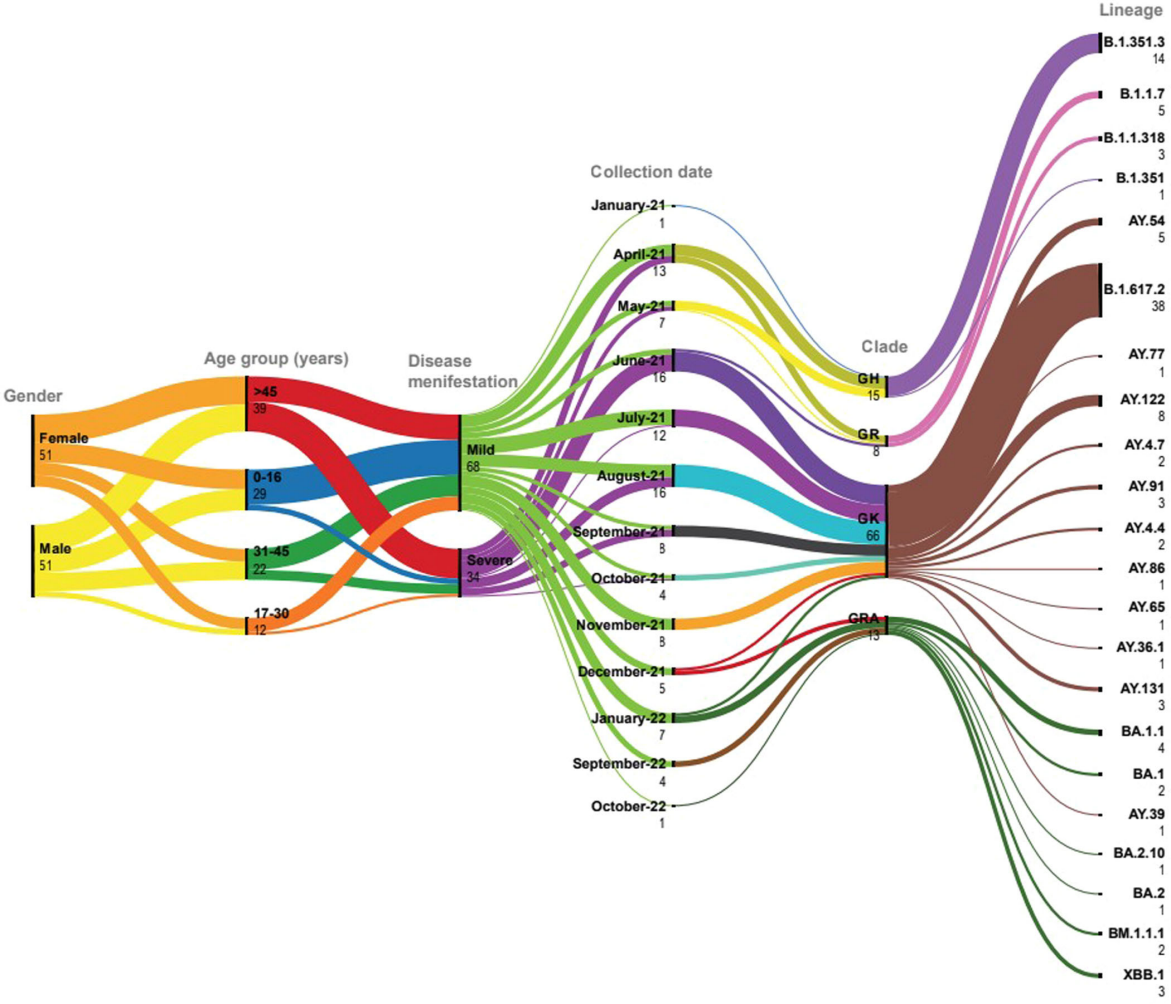
The distribution of different types of severe acute respiratory syndrome coronavirus 2 (SARS‐CoV‐2) in the Chittagong region between January 2021 and October 2022. The group title was presented at the top, and the number of genomes under different classes was shown aside.

The presentation of our data over time in an alluvial diagram showed some noteworthy findings. Regarding disease manifestations, the severe outcome was not recorded after November 2021 in our study. Between January 2021 and June 2021, samples (*n* = 102) were clustered in GH (*n* = 15) and GR (*n* = 8) clades. Subsequently, GK clade was introduced in diversity in June 2021 and continued its dominance until January 2022. In December, we noticed that SARS‐CoV‐2 classified in GRA clade, was circulating until we finished the study in October 2022 (Figure 2).

**Figure 3:**
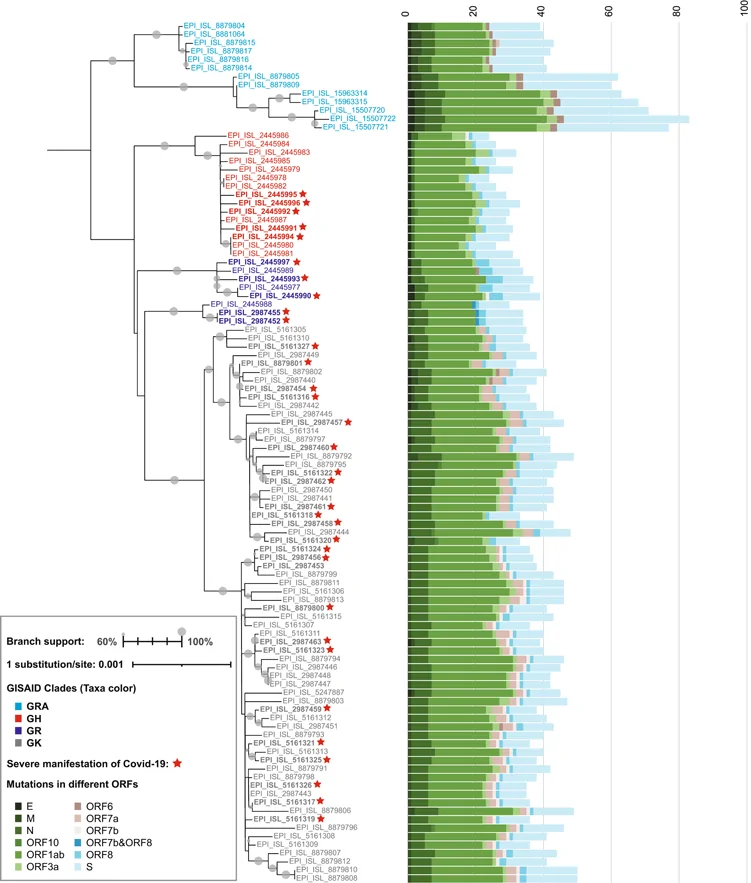
Genomic diversity of found in 102 severe acute respiratory syndrome coronavirus 2 (SARS‐CoV‐2) clinical samples in the Chittagong region (*N* = 102). A Maximum likelihood phylogenetic tree was generated using RaxML analysis of alignments of 102 clinical samples of SARS‐CoV‐2.^21^ The legend for subfamily and inter‐subfamily support and support values within subfamilies is shown in an inset box. Support values less than 60% maximum likelihood bootstrap are not displayed. The length of branches in the tree corresponds to the number of substitutions per site (as indicated by the scale bar). The taxa are color‐coded according to their GISAID clades, and those labeled in bold with a red star symbol are associated with severe disease manifestation. A stacked bar chart represents each sample's number of mutations in different ORFs. Tree annotation was carried out with iTOL.^27^

SARS‐CoV‐2 genomes sequenced between December 2021 and October 2022 belonged to GRA clade and manifested mild COVID‐19. We screened the genome sequences through phylogenetic analysis and found that GRA clade clustered separately from other clades of SARS‐CoV‐2. A high genomic mutation burden could be attributed to this separate clustering. Our study's overall mean mutation burden in all other clades (GK, GH, and GR) was 38.134 compared to the Wuhan reference genome. The burden was 1.5‐fold higher in the GRA clade (mean mutations found 56.07 per genome), all caused by mild disease (Figure 3). Most of the mutations were found in the S and ORF1ab segments. Interestingly, the burden of mutations in the GRA clade was found mainly due to the accumulation of mutation in the S segment; 22.15 mutations per S segment of the GRA clade versus 9.39 in others (Figure 3).

### 3.3 Mutation profile

A total of 4123 different mutations were found throughout different genomic segments of SARS‐CoV‐2 in our study (Supporting Information S1: Table S1). Most of these mutations were missense (67.5%), and the most affected genomic elements with this mutation type were ORF1ab (1083), S (886), N (342), ORF3a (122), ORF7a (145), and M (9). Synonymous mutations were the second most (755, 18.31%) prevalent genomic mutation in this cohort. We also observed upstream gene variants (194), disruptive inframe deletion (105), conservative inframe mutations (96), downstream gene variants (83), frameshift variants (68), and others (40) (Table 2). Notably, all the downstream gene variants were found within the S gene, and 67 out of 68 frameshift mutations were located in N. Conservative inframe deletions were primarily found in ORF8 (68.75%), and the rest were in ORF1ab (31.25%).

**Table 2:** Unique mutations of SARS‐CoV‐2 in mild and severe manifestations of COVID‐19.

Gene/ORF	Mutation	Frequency	Remarks
*Unique mutations in the mild manifestation of COVID‐19*			
ORF1ab	I5967V	13	Located in the NSP (nonstructural proteins)−3 encoding region of ORF1a, both are nonpolar, hydrophobic, aliphatic AA
	P3395H	13	Located in the NSP2 region, having a role in vesicle trafficking, nonpolar, hydrophobic to polar, essential, hydrophilic AA
	R5716C	7	Located in the NSP13 region encoding helicase enzyme, both are polar, hydrophilic AA
	G1307S	7	Located in the NSP3 region inhibiting IFN signaling and blocking host innate immune response, both are polar, hydrophilic AA
	L3027F	7	Located in the NSP4 region contributing to double‐membrane vesicle (DMV) formation, aliphatic to aromatic AA
	L3201F	7	Located in the NSP4 that contributes to transmembrane scaffold protein structure, aliphatic to aromatic AA
	S135R	7	Located in the NSP1 region, playing a role in cellular degradation promotion, hydroxylic to basic AA
	T3090I	7	Located in the NSP2 region of ORF1a, having a function of vesicle trafficking, polar, hydrophilic to non‐polar, hydrophobic AA
	T6564I	7	Located in the NSP15 region encoding NendoU enzyme, polar, hydrophilic to non‐polar, hydrophobic AA
	T842I	7	Located in the NSP2 region binding to prohibition protein, polar, hydrophilic to non‐polar, hydrophobic AA
	A2710T	6	Located in the NSP3 region of ORF1a, non‐polar, hydrophobic to polar, hydrophilic AA
	I3758V	6	Located in the NSP6 region contributing to the structure of DMV, both are non‐polar, hydrophobic, aliphatic AA
	L3674_G3676del	6	Located in the NSP3 region of ORF1a
	K856R	6	Located in the NSP2 region that binds to prohibition proteins, both are polar, hydrophilic, basic AA
	S2083_L2084delinsI	6	Located in the NSP2 region of ORF1a
ORF6	D61L	**7**	Located in ORF6, polar aliphatic to non‐polar aliphatic AA
	W27L	2	Located in ORF6, aromatic to aliphatic
	E54*	1	ORF6 interacts with the nsp8 protein coded by SARS‐CoV‐2, and it can increase infection during early infection at a low multiplicity with an increase in RNA polymerase activity.^28^
	P57L	1	Located in ORF6, both are hydrophobic aliphatic, affecting protein function
ORF7a	P84S	2	Located in ORF7a, Hydrophobic to Hydrophilic
	P99S	2	Located in ORF7a, Hydrophobic to Hydrophilic
	P45L	1	Located in ORF7a, both are hydrophobic aliphatic, shown to affect disease^29^
	V104F	1	Located in ORF7a, Aliphatic to Aromatic, deleterious effect on protein
	V82S	1	Located in ORF7a, Hydrophobic to Hydrophilic
ORF8	G8V	1	It is located in the ORF8. Polar and flexible to non‐polar and branched‐chain amino acid, which could help boost host immune response.^30^
S	N679K	13	It is located upstream of the S1/S2 cleavage site—neutral, polar amino acid to Basic, polar amino acid.
	N764K	13	It is located downstream of the S1/S2 cleavage site. Neutral, polar amino acid to Basic, polar amino acid
	N969K	13	It is located in the heptad repeat (HR1) of the S2 region. Neutral, polar amino acid to Basic, polar amino acid
	D796Y	13	It is located upstream of the S1/S2 cleavage site. Acidic, aliphatic & polar amino acid to aromatic, polar amino acid
	Q954H	13	It is located in the heptad repeat (HR1) of the S2 region. Neutral, polar amino acid to Basic, polar amino acid
	H655Y	13	It is located in Subdomain 2 (SD2) of the S1 region. Basic, polar amino acid to Neutral, polar amino acid
	G339D	8	It is located in the receptor binding domain (RBD) of the S1 region. Polar and flexible amino acid to Neutral, polar amino acid
	Q498R	7	It is located in the receptor binding domain (RBD) of the S1 region. Neutral, polar amino acid to Basic, polar amino acid
	E484A	7	It is located in the receptor binding domain (RBD) of the S1 region. Acidic, polar amino acid to neutral, non‐polar amino acid
	ST477NK	7	It is located in the receptor binding domain (RBD) of the S1 region—neutral polar amino acids to neutral and basic polar amino acids.
	Y505H	7	It is located in the receptor binding domain (RBD) of the S1 region. Neutral, polar amino acid to Basic, polar amino acid
	A67_V70delinsVI	6	It is located in the N‐terminal domain (NTD) of the S1 region. All of them are non‐polar amino acid
	N440K	6	It is located in the receptor binding domain (RBD) of the S1 region. Neutral, polar amino acid to Basic, polar amino acid and the mutation is reported to be associated with immune escape
	N856K	6	It is located in the connecting region (CR) of the S2 region. Neutral, polar amino acid to Basic, polar amino acid
	G446V	6	It is located in the receptor binding domain (RBC) of the S1 region. Both are neutral, polar amino acid
	L24_A27delinsSer	6	
	L981F	6	Located in the heptapeptide repeat sequence‐1 of the S1 subunit. A non‐polar and aliphatic amino acid changed to aromatic amino acid
	T19I	6	Located in the N‐terminal domain of the S protein. A neutral and polar amino acid changed to nonpolar aliphatic amino acid
	T547K	6	Located in between heptapeptide repeat sequences 1 and 2. A neutral and polar amino acid changed to a positively charged amino acid
	N460K	5	Located in the receptor binding domain of the S1 subunit. A neutral and polar amino acid changed to a positively charged amino acid
	F486S	5	Located in the receptor binding domain of the S1 subunit. An aromatic amino acid changed to polar uncharged amino acid
	F490S	5	Located in the receptor binding domain of the S1 subunit. An aromatic amino acid changed to neutral and polar amino acid
*Unique mutations in the severe manifestation of COVID‐19*			
ORF1ab	A4489V	2	Located in nonstructural protein 2 of ORF1ab. Both are non‐polar and aliphatic amino acids
	S538L	2	Located in nonstructural protein 2 of ORF1ab. Neutral and polar amino acid to non‐polar aliphatic amino acid
	A1049V	1	Located in nonstructural protein 3 of ORF1ab. Both are non‐polar and aliphatic amino acids
	A3523V	1	Located in nonstructural protein 5 of ORF1ab. Both are non‐polar and aliphatic amino acids
	A6296V	1	Located in nonstructural protein 14 of ORF1ab. Both are non‐polar and aliphatic amino acids
ORF7a	G38V	1	Located in the luminal domain of the type‐I transmembrane protein. Both are non‐polar and aliphatic amino acids
	L17F	1	Located in the luminal domain of the type‐I transmembrane protein. Non‐polar and aliphatic amino acid to Aromatic amino acid
ORF7b	T40I	12	ORF7b is an integral membrane protein. Anti‐7b protein was found. A neutral and polar amino acid to non‐polar aliphatic amino acid
	M1fs	1	Frameshift Mutation
ORF8	D35Y	1	Located in the ORF8 that downregulates the MHC‐I expression. A negatively charged amino acid to aromatic amino acid.
	S54L	1	Located in the ORF8 that downregulates the MHC‐I expression. Neutral and polar amino acids to non‐polar aliphatic amino acids.
S	V1228L	2	Located in the TM domain of the S2 subunit. Both are non‐polar and aliphatic amino acids.
	A67V	1	Located in the N‐terminal sequences of the S protein. Both are non‐polar and aliphatic amino acids
	Q173H	1	Located in the N‐terminal sequences of the S protein. A neutral and polar amino acid changed to a positively charged amino acid.
	Q677H	1	Located in between S1 and S2 subunits. Neutral and polar amino acid to positive amino acid
	E554Q	1	Located in between S1 and S2 subunits. A negatively charged amino acid changed to neutral and polar amino acid
	L141_Y144delinsF	1	Located in the N‐terminal domain of the S1 subunit. Non‐polar and aliphatic amino acids are replaced by aromatic amino acid
	L54F	1	Located in the N‐terminal domain of the S1 subunit. A non‐polar and aliphatic amino acid changed to aromatic amino acid
	V143fs	1	Frameshift mutation

Abbreviations: COVID‐2019, coronavirus disease 2019; SARS‐CoV‐2, severe acute respiratory syndrome coronavirus 2.

*Introduction of the Stop codon.

### 3.4 Determinants of genetic diversity in COVID‐19 outcomes

**Figure 4:**
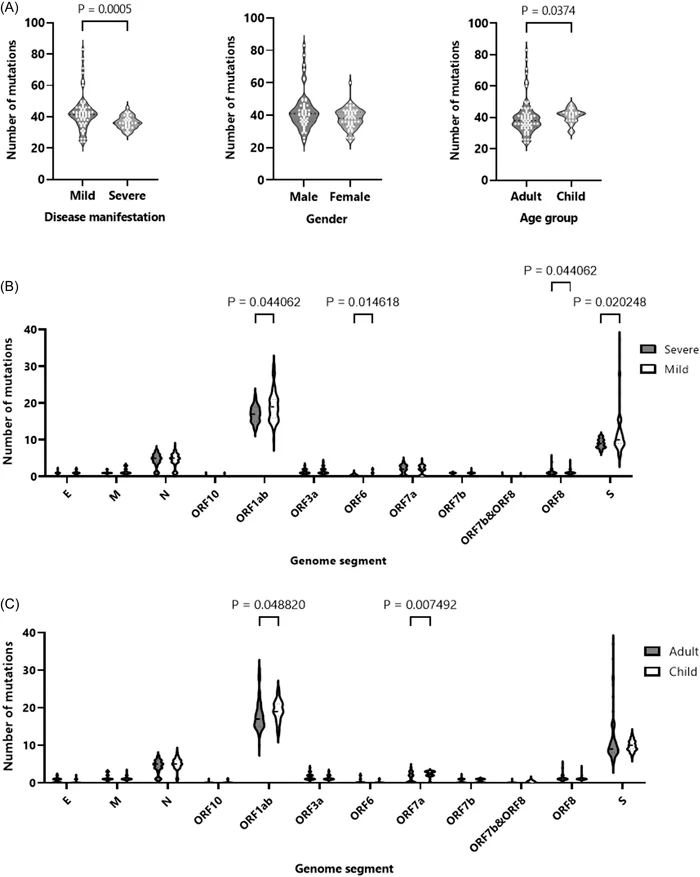
Correlation of COVID‐19 clinical outcome: gender and age group with the frequency of SARS‐CoV‐2 mutations (A) and determinants of SARS‐CoV‐2 genetic divergence in COVID‐19 disease outcomes (B) and age groups (C). COVID‐2019, coronavirus disease 2019; SARS‐CoV‐2, severe acute respiratory syndrome coronavirus 2.

To find out the genetic determinants of the outcome of the disease caused by SARS‐CoV‐2, we analyzed the correlation between mutation frequency with three key variables: disease manifestations, gender and age of the patients. We found no correlation between gender and mutation frequency (Figure 4). Nevertheless, the mutation frequency was significantly correlated with the disease manifestation (Mann–Whitney *U* = 673; *p* = .0005) (Figure 4A). Furthermore, the median number of mutations per genome in mild patients was significantly higher (41.5), whereas 36 were among the genome samples isolated from the severely affected patients. In the case of genetic alterations among adult versus children, the median of children is significantly higher than that of adults (38 vs. 42; Mann–Whitney *U* = 797.5; *p* = .0374).

Multiple unpaired, nonparametric Mann–Whitney tests were done among mutations in different gene elements (Figures 4B,C). The results indicated that the five different gene segments, namely, ORF1ab, ORF6, ORF7a, ORF8, and S significantly differ regarding genetic divergence in mild manifestations of COVID‐19.

To spot the specific mutations behind the disease outcomes reported in the previous section, we analyzed the unique mutations among these genetic segments and predicted their possible role in viral fitness. We found that the number of mutations in mild cases was twice as many as in severe cases (414 vs. 193; Supporting Information S1: Figure S1).

**Figure 5:**
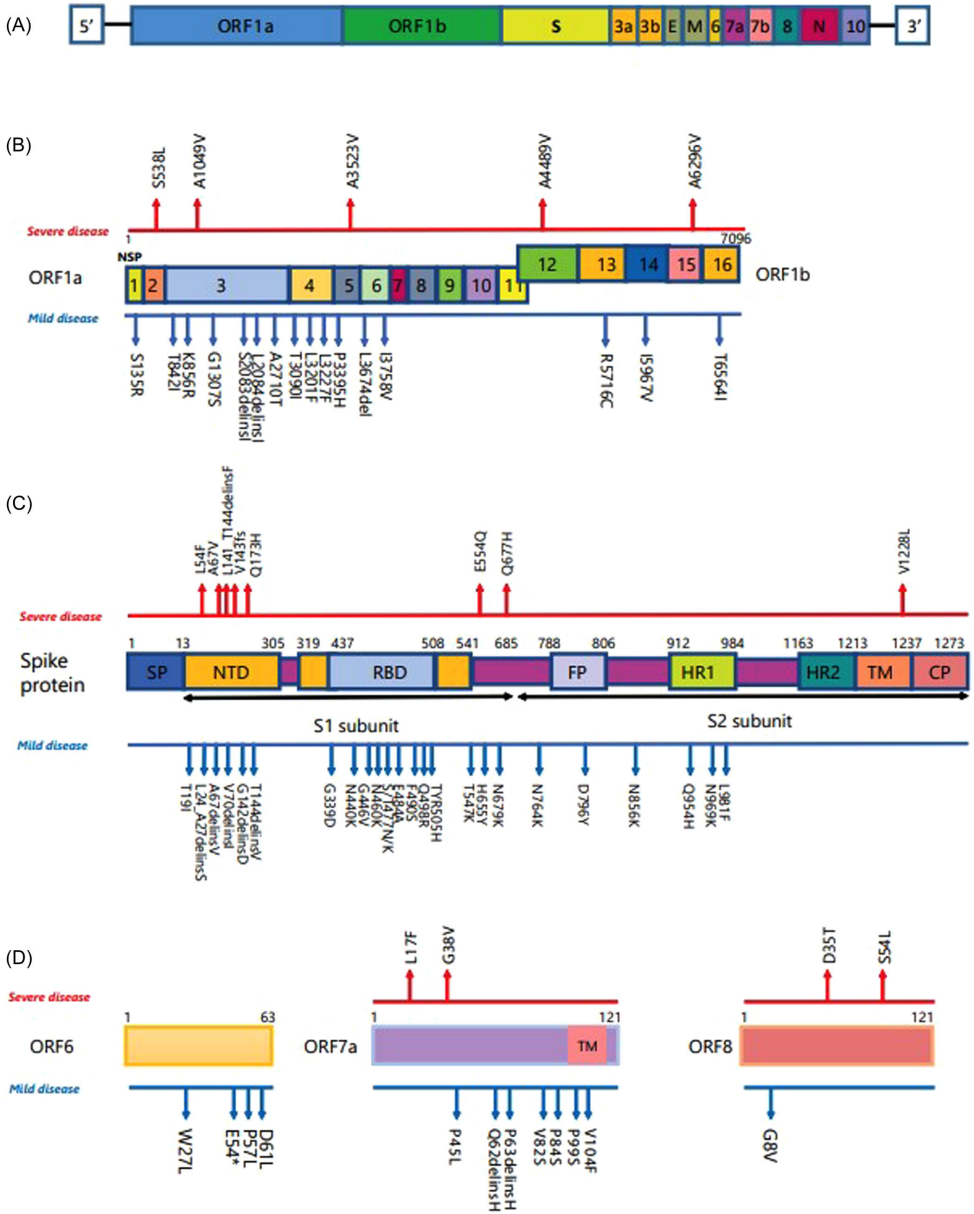
Distribution of unique mutations over the severe acute respiratory syndrome coronavirus 2 (SARS‐CoV‐2) genome (A; adopted from Koyama et al.^31^) from severe and mild samples. Here we presented the genome segments found to have significant differences in mutation burden, namely ORFab (B), Spike protein (C), and ORF6, ORF7a, and ORF8 (D). The relative position of the mutation was presented in red (−) and blue (−) for samples with severe and mild disease, respectively.

Notably, the burden of unique mutations in the mild COVID‐19 cases was more than four times higher than in the severe cases (286 vs. 65; Supporting Information S1: Figure S1). The burden of the missense (a point mutation where a single nucleotide was changed to cause a substitution of a different amino acid), nonsense mutations (introduces a stop codon to the gene sequence) and indels (insertion and deletions) were analyzed and presented in Figure 5 and Supporting Information S1: Figure S2. The consequences of the changes and their frequency in our data set were described in Table 2.

The burden of unique mutations was significantly higher in mild patients than in severe counterparts by type and frequency. We found the type of unique mutations among mild patients more than three times the severe patients in ORF1ab (16 vs. 5) and spike protein (24 vs. 8). We only reported four unique mutations in the small viral protein ORF6 among mild patients. The pattern was unchanged from ORF7a, 7 in mild versus 2 in severe samples. The only exception was ORF8, where we saw two unique mutations in severe samples and one in mild (Figure 5).

The most frequent unique mutations in mild cases were analyzed. For ORF1ab were I5967V and P3395H, with a frequency of 13. In spike protein, we recorded 13 appearances for six mutations, namely N679L, N764L, N969L, D796Y, Q954H, and H655Y. The most frequent mutation in ORF6 was D61L, with a frequency of 6 (Table 2). In severe samples, almost all mutations appeared one to two times except Thr40Ile of ORF7b with a frequency of 12 (Table 2).

**Figure 6:**
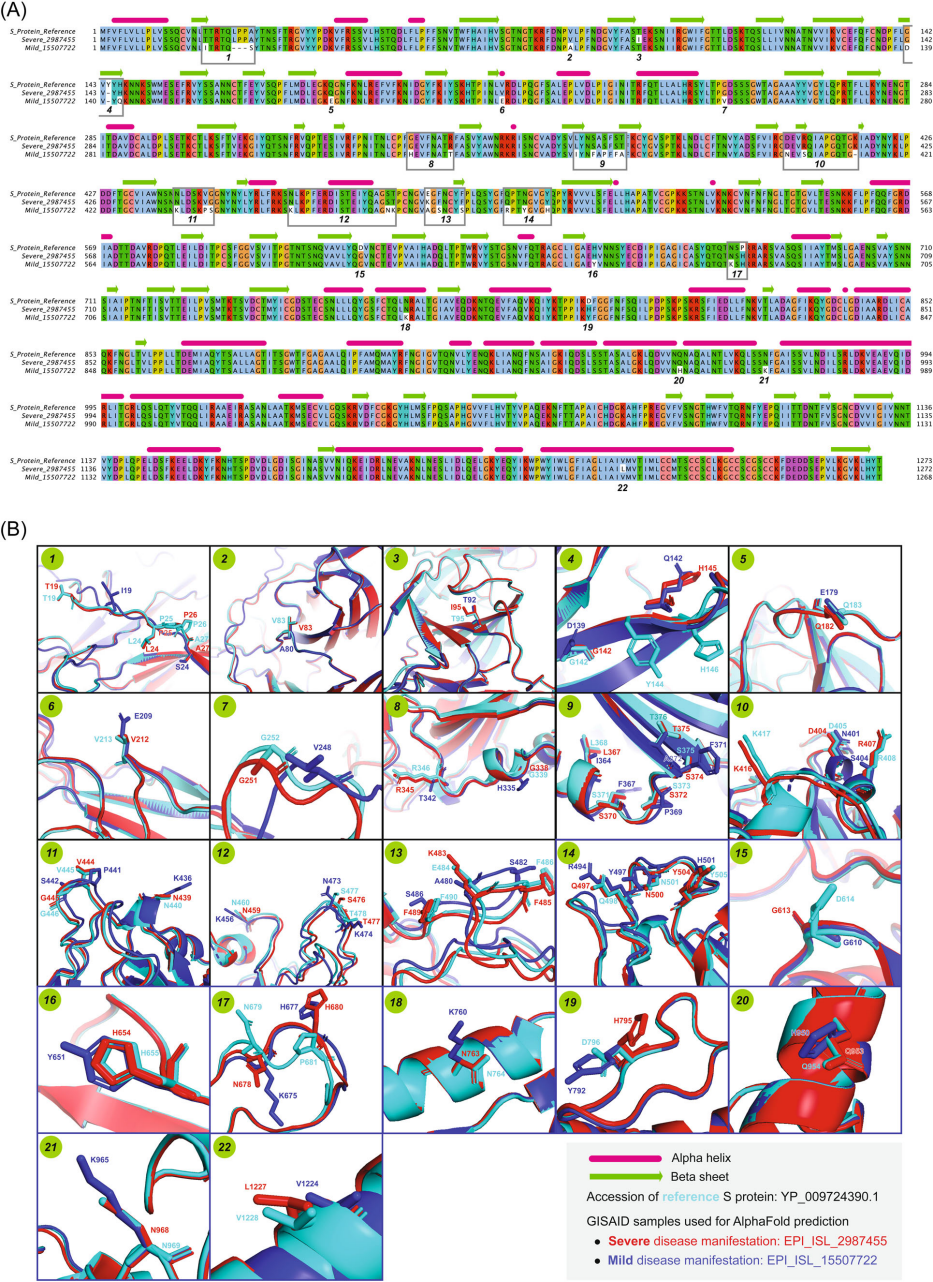
Structural divergence of spike protein in representative highly mutated SARS‐CoV‐2 genome from severe and mild COVID‐19 patients. The sequence alignment (A) and superimposed structures (B) present the structural basis of accumulated mutations. The mutated amino acids are shown as sticks. COVID‐2019, coronavirus disease 2019; SARS‐CoV‐2, severe acute respiratory syndrome coronavirus 2.

To demonstrate the structural significance of the accumulated mutations on the SARS‐CoV‐2 genome, we further presented the mutations of the S protein, as this protein was seen more than twofolds in mild cases, we found in our study by comparing the predicted structure using AlphaFold2 (Figure 6 and Supporting Information S1: Figure S3). We have seen the accumulation of aromatic rings in alpha helices and beta sheets, shortening the loops, replacements of simple amino acids, and turning throughout the protein (Figure 6B). The structural divergence of S proteins from the mild COVID‐19 patient portrayed insights into how the mutations lead to the less virulent form of the virus.

## 4 DISCUSSION

The SARS‐CoV‐2 virus, due to the lack of proofreading activity of the RNA‐dependent RNA polymerase, has high mutation rates that may have important effects on the pathogenicity and transmissibility of the virus.^27^ Identifying genome variations of SARS‐CoV‐2 and their relationships with viral infectivity or severity of COVID‐19 is crucial for controlling and surveying the evolution of the pandemic.^31^ In addition, the mutation rate of SARS‐CoV‐2 determines the evolution of this virus and the risk of emerging infectious diseases.^28^ This study analyzed the relationship between disease outcomes, demographic characteristics and mutation burden and its consequences on the structural features of the essential viral constituents of the SARS‐CoV‐2 genome isolated in the southern part of Chittagong.

Though this study enrolled 102 COVID‐19 patients and found that gender and previous history were not significantly related to the disease severity, the number of males with mild symptoms was higher than that of women (Table 1). This finding aligns with previous studies that males are more susceptible to SARS‐CoV‐2 than that of women.^29^ On the other hand, the number of men with severe disease symptoms was less than that of women. This study is opposite to some of the previous findings that found the number of men who died was significantly higher than that of women.^30^, ^32^ Possibly, the low number of data sets impacted our results. In this RT‐PCR‐based detection study, COVID‐19 reinfection was not associated with disease severity. A similar type of detection by Hall et al.^33^ reported that a prior SARS‐CoV‐2 infection lowered the disease risk with the following infection. They also reported acquired immunity development due to the first SARS‐CoV‐2 that could sustain up to the next 7 months.^33^, ^34^ Regardless of the gender variable, some studies found reinfection with or without some mutations in the next viral genome, making the disease either mild or severe.^34^

We found that the disease was significantly more severe among adults than the younger ones. Other studies also found that age is an obvious risk factor for COVID‐19 disease.^35^ Ageing causes differences in innate and adaptive immunity. Children have a more rigorous mucosal innate immune response against SARS‐CoV‐2 due to having their gut microbiota more intact than adults. Besides, ageing can cause higher expression of the human ACE2, a Spike protein receptor. Thus, ageing might be a replication enhancer of SARS‐CoV‐2.^36^

Our study suggests that previous vaccination reduced the disease manifestation on reinfection. A similar observation was found in another study, which stated that the probability of reinfection was significantly lower among vaccinated individuals than the nonvaccinated.^37^, ^38^ However, it was also crucial to consider if variants, like Delta and Omicron, were specific to the vaccine regimen in this area during the study.^38^ Also, the doses and duration of the vaccination protection were considered additional factors.

Our study also found a significant relationship between severe COVID‐19 and the primary risk factors of COVID‐19 like hypertension, diabetes, and renal diseases (Figure 1). Increased systolic and diastolic blood pressure was correlated with increased ACE2 activity in blood.^39^, ^40^ A study found a higher concentration of ACE2 in the significantly higher systolic and diastolic blood plasma than in the control group.^41^ However, it is yet to be determined if plasma ACE2 directly helps SARS‐CoV‐2 to replicate, but ACE2 release could cause endothelial dysfunction and hyperinflammation, and consequently, COVID‐19 might get progressively severe.^42^

Our sample cohort has shown a correlation between the mild manifestation of disease and mutation burden. When the vaccine administration was at its peak in Bangladesh, we did not see any severe manifestation of the disease (Figure 2). We found that the strain that dominated during that time belonged to the GRA clade, which bore more than twofold mutations per S gene segment of the SARS‐CoV‐2 genome (Figure 3). It was estimated that the SARS‐CoV‐2 genome accumulates two‐single nucleotide mutations every month.^43^ Our report strengthened the estimates of mutation accumulation in the SARS‐CoV‐2 genome over time.

A higher frequency of missense mutations in our study cohort is consistent with previous reports, indicating that SARS‐CoV‐2 exhibits high genetic variability Table S1). In our study, the most occurring mutations were missense mutations followed by synonymous and indel mutations. A previous study also reported that most occurring mutations were missense, synonymous, start/stop codons, and mutations in UTR regions.^44^, ^45^ One potential explanation for the higher frequencies of missense mutations could be ORF1ab and S genes' susceptibility to missense mutations. Also, these mutations might have functional importance in the virus's replication and entry mechanisms, respectively. On the other hand, synonymous mutations might not have any role in protein folding but have profound effects on mRNA folding, stability, and translation for viral fitness under solid selection pressure.^46^

Our findings suggest that increased genetic variability of the virus is significantly associated with less severe disease outcomes (Figure 4). This variability could be due to a higher viral replication and mutation rate in patients with mild disease, leading to increased genetic diversity in the viral population. Alternatively, the host's immune response might play a role in shaping the viral mutation patterns. The immune system might exert selective pressure on the virus, leading to the emergence of different viral variants with varying mutation frequencies.^47^

The significant difference in mutation frequency between children and adults is a notable finding that warrants further investigation. The immune response and viral replication dynamics in children may differ from adults, leading to differences in mutation frequency. Children may have a more robust and intact immune response against SARS‐CoV‐2, resulting in increased viral clearance and reduced replication, which in turn may affect the mutation patterns of the virus for better fitness.^48^ The lack of a significant correlation between mutation frequency and gender in our study cohort suggests that gender may not be a substantial determinant of SARS‐CoV‐2 mutation patterns. However, further studies with larger sample sizes could give more insight into this finding and explore potential gender‐specific differences in viral mutation patterns.

We found frequent mutations in mild and severe cases (Figures 5, 6, and Table 2). Some of them are found in 20% of mild cases. Among them, we frequently found spike protein mutations (N764K, N969K, D796Y, Q954H, N679K, and H655Y), which were also reported in previous studies.^12^, ^49^, ^50^, ^51^ Among them, N679K was reported to decrease viral virulence by decreasing spike protein yield and potentially helping host immunity.^50^

To conclude, concurrent detailed and large‐scale studies are necessary to validate these findings. The study's major limitation is the sample size (*n* = 102), which was deliberate due to limited funding and high cost of sequencing. Moreover, during the study period, retrospective data and samples were collected from only two hospitals in Chattogram, Bangladesh.

Overall, different public health measures taken and a good pace of vaccination during the study period in Bangladesh might impose selective pressure on the virus to get mutated. The pattern of difference in mutation profiles between mild and severe manifestations, based on the findings of our study, makes it claimable that acquiring mutations might cause SARS‐CoV‐2 to lose its virulence to some extent.

## 5 CONCLUSION

This study investigated SARS‐CoV‐2 infected patients' clinical outcomes and viral genomic characteristics in the Chattogram division, Bangladesh. We reported that the disease severity was significantly higher in adults than in children. The two most prevalent comorbidities in hospitalized cases were hypertension and diabetes. In comparison to the other clades (GH, GK, and GR), the mutation burden was much higher in GRA (1.5 folds) in case of mild symptoms. Moreover, we observed many unique mutations in S protein in mild cases compared to severe, and homology modeling revealed that those might cause more folding in the protein's alpha helix and beta sheets. In addition, our findings suggest that the mutations in the GRA clade may have interfered with viral transmissibility and immune evasion potential and that mild cases may harbor more genetic diversity than previously thought. Therefore, these results have important implications for the surveillance and control of COVID‐19 in Bangladesh and beyond. Further studies are needed to confirm the mutations' functional effects and monitor the emergence of new variants.

## AUTHOR CONTRIBUTIONS

**Md. Mahbub Hasan**: Conceptualization; data curation; methodology; formal analysis; resources; software; validation; visualization; writing—original draft. **Chayan Kumar Saha**: Data curation; formal analysis; methodology; software; validation; visualization. **H. M. Hamidullah Mehedi**: Data curation; supervision; project administration; investigation; methodology. **Kallyan Chakma**: Conceptualization; data curation; investigation; project administration; resources. **Asma Salauddin**: Conceptualization; data curation; investigation; methodology; validation. **Md Shakhawat Hossain**: Data curation; investigation; methodology; resources; writing—original draft. **Farjana Sharmen**: Investigation; project administration; writing—original draft. **S. M. Rafiqul Islam**: Conceptualization; supervision; writing—review & editing. **Afroza Akter Tanni**: Validation; writing—original draft. **Farhana Yasmin**: Data curation; investigation. **Al‐Shahriar Akash**: Data curation; investigation. **Mohammad Enayet Hossain**: Data curation; investigation; methodology. **Mojnu Miah**: Data curation; investigation; methodology. **Sanjoy Kanti Biswas**: Conceptualization; methodology; project administration. **Nahid Sultana**: Conceptualization; investigation; project administration. **Adnan Mannan**: Conceptualization; investigation; project administration; methodology; supervision; resources; writing—review & editing.

## CONFLICT OF INTEREST STATEMENT

The authors declare no conflict of interest.

## ETHICS STATEMENT

Ethical approval was granted from Institutional Review board (IRB) of 250 bedded General Hospital, Chattogram. Oral and written consent from the patients were taken.

## Supporting information

Supporting information.

## Data Availability

The data collected during this study will be available from the corresponding author upon reasonable request.
