# Supplementary material for: Genetic determinants of SARS‐CoV‐2 and the clinical outcome of COVID‐19 in Southern Bangladesh
Source: Immun Inflamm Dis. 2024 Feb 6;12(2):e1171. doi: 10.1002/iid3.1171 (PMC10845815; doi:10.1002/iid3.1171)
Supplement: Supplementary file 1 — Supporting information. [file IID3-12-e1171-s001.docx]

**Supplementary figure and table**


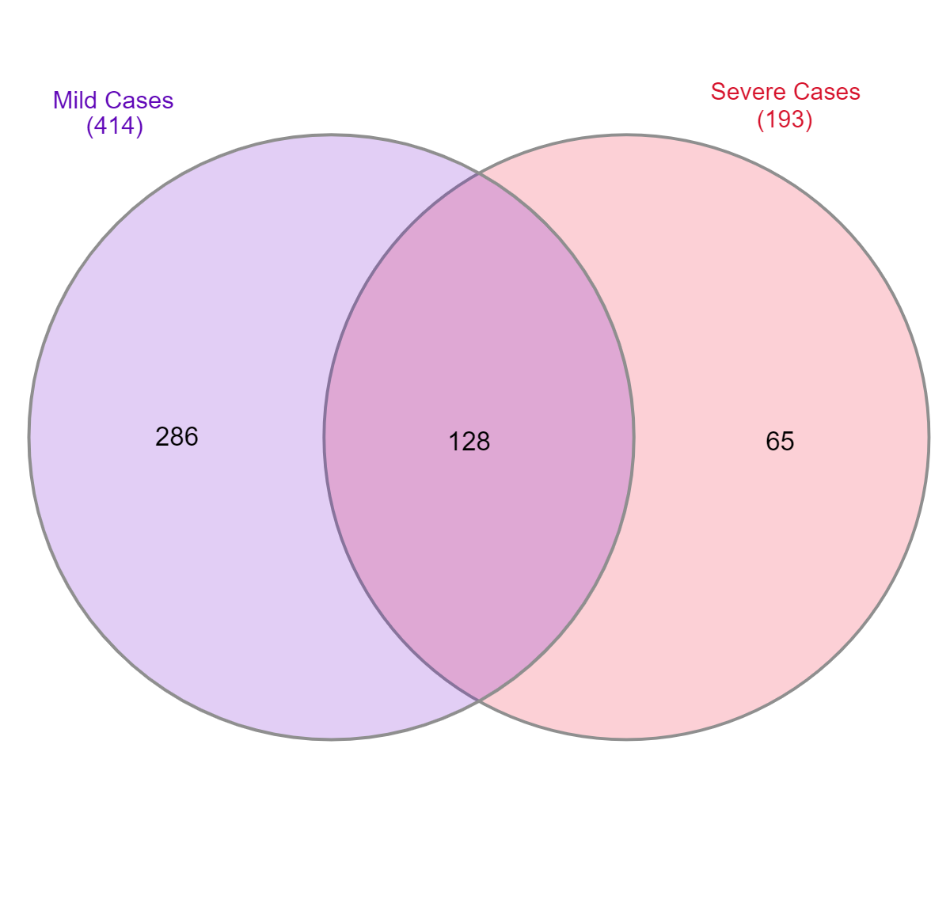


**Figure S1.** **The distribution of SARS-CoV-2 mutations between mild and severe COVID-19 outcomes**.


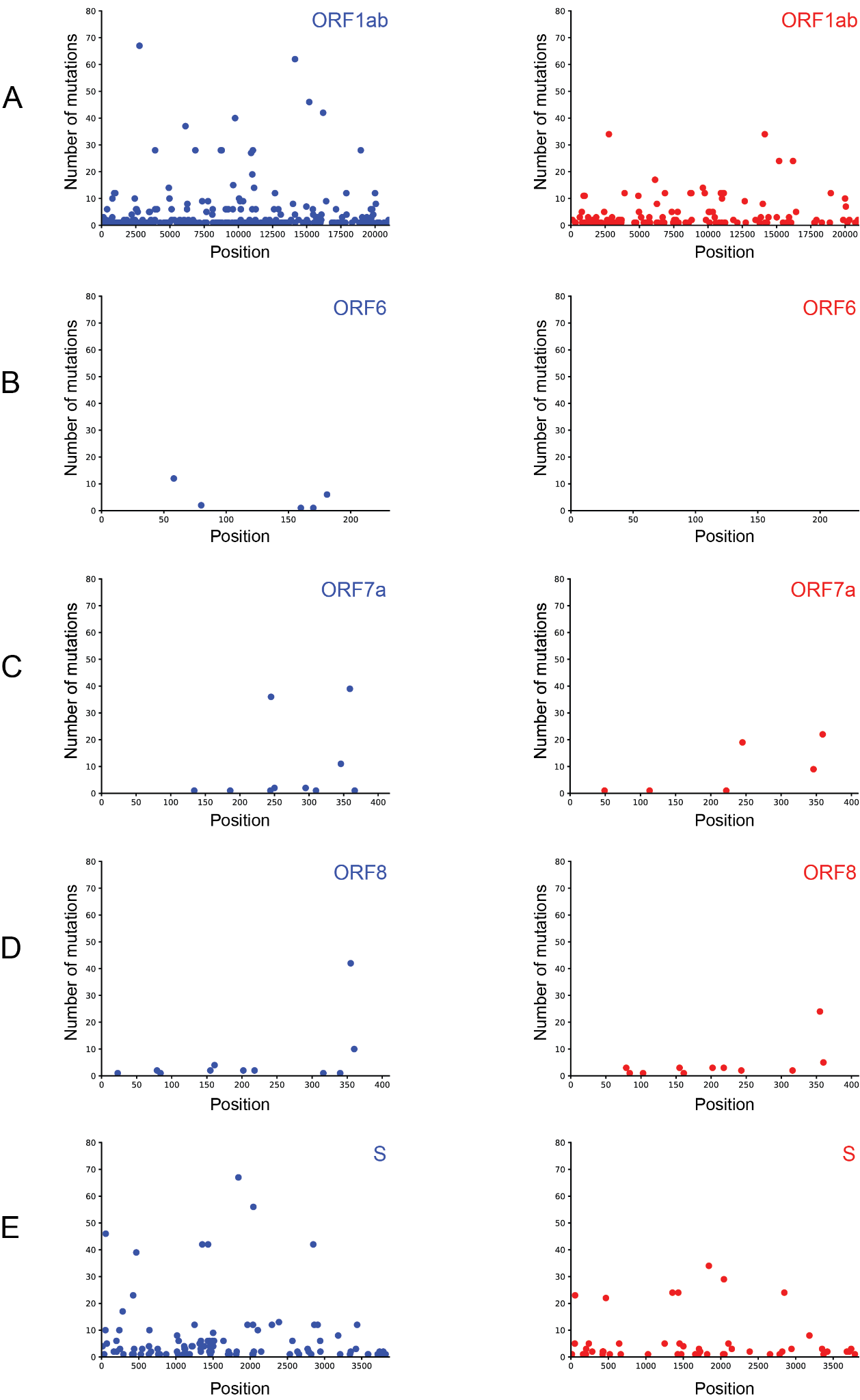


**Figure S2:** **Distribution of mutations of SARS-CoV-2** in ORF1ab (A), ORF6 (B), ORF7a (C), ORF8 (D) and protein S (E) from mild and severe patients of Chittagong.


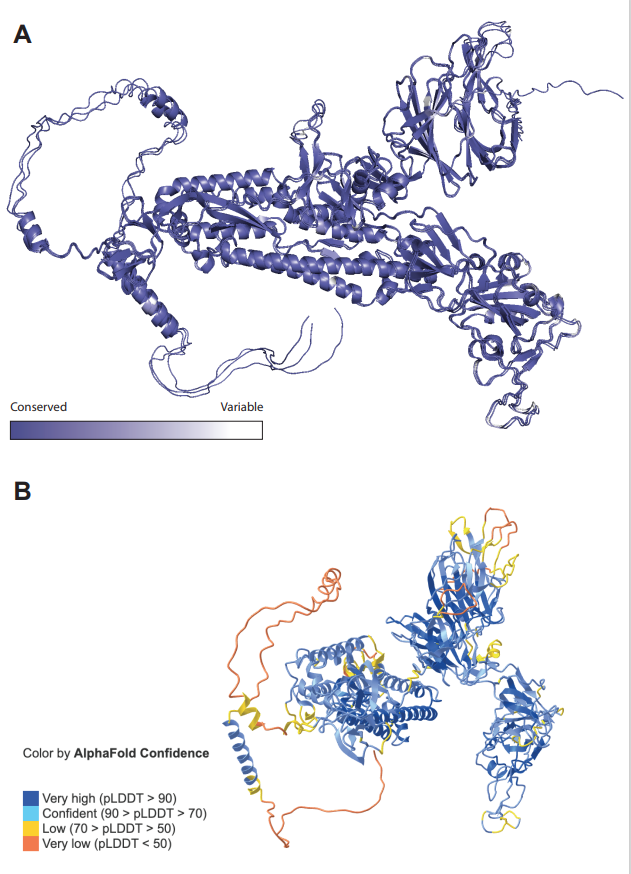


**Figure S3:** **The superposition of representative S protein from mild and severe patients**, a reference from Wuhan (A) and the homology modelling confidence values throughout the S protein (B).

| **Table S1. Types of mutation found in all SARS-CoV-2 samples (n=102).** | | | | | | | | | |
| --- | --- | --- | --- | --- | --- | --- | --- | --- | --- |
| **Genome segment** | **Missense variant** | **Synonymous variant** | **Upstream gene variant** | **Disruptive inframe deletion** | **Conservative inframe deletion** | **Downstream gene variant** | **Frameshift variant** | **Others** | **Total** |
| **ORF1ab** | 1083 | 579 | 171 | 12 | 30 | 0 | 0 | 0 | 1875 |
| **S** | 886 | 64 | 0 | 76 | 0 | 83 | 0 | 15 | 1124 |
| **N** | 342 | 21 | 16 | 16 | 0 | 0 | 67 | 0 | 462 |
| **ORF3a** | 122 | 31 | 0 | 0 | 0 | 0 | 0 | 0 | 153 |
| **ORF7a** | 145 | 1 | 4 | 1 | 0 | 0 | 0 | 1 | 152 |
| **M** | 99 | 17 | 0 | 0 | 0 | 0 | 0 | 0 | 116 |
| **ORF8** | 14 | 19 | 0 | 0 | 66 | 0 | 0 | 17 | 116 |
| **E** | 40 | 9 | 0 | 0 | 0 | 0 | 0 | 0 | 49 |
| **ORF7b** | 41 | 0 | 0 | 0 | 0 | 0 | 1 | 1 | 43 |
| **ORF6** | 10 | 13 | 0 | 0 | 0 | 0 | 0 | 1 | 24 |
| **ORF10** | 0 | 1 | 3 | 0 | 0 | 0 | 0 | 2 | 6 |
| **ORF7b & ORF8^a^** | 0 | 0 | 0 | 0 | 0 | 0 | 0 | 3 | 3 |
| **Total** | 2782 | 755 | 194 | 105 | 96 | 83 | 68 | 40 | 4123 |
| ^a^ gene fusion between two segments of the genome. | | | | | | | | | |
